# Supplementary material for: Reductions to health-related quality of life associated with cigarette use, e-cigarette use, and depression among US adults
Source: medRxiv. 2026 May 27:2026.03.19.26348841. Originally published 2026 Mar 23. Preprint. [Version 2] doi: 10.64898/2026.03.19.26348841 (PMC13042109; doi:10.64898/2026.03.19.26348841)
Supplement: Supplement 1 [file media-1.pdf]

Supplement

Table 1. Utility scores by cigarette use, depression, and e-cigarette use status - men ages 18+

	Depressed		Not depressed	
Age	Use E-cigarette	Not Use E-cigarette	Use E-cigarette	Not Use E-cigarette
Never smoking				
18-24	0.799 (n=781)	0.805 (n=2995)	0.832 (n=2525)	0.843 (n=16677)
25-44	0.781 (n=659)	0.798 (n=7684)	0.824 (n=1991)	0.827 (n=45413)
45-64	0.759 (n=135)	0.773 (n=7589)	0.795 (n=38)	0.818 (n=57979)
65+	0.724 (n=41)	0.7405 (n=5540)	0.792 (n=185)	0.78 (n=55421)
Current smoking				
18-24	0.799 (n=360)	0.799 (n=241)	0.829 (n=699)	0.843 (n=618)
25-44	0.771 (n=1133)	0.771 (n=2657)	0.816 (n=2208)	0.821 (n=8309)
45-64	0.757 (n=552)	0.717 (n=3844)	0.797 (n=1159)	0.797 (n=12512)
65+	0.703 (n=100)	0.7075 (n=1621)	0.773 (n=405)	0.776 (n=8408)
Former smoking				
18-24	0.797 (n=370)	0.797 (n=231)	0.829 (n=682)	0.843 (n=746)
25-44	0.776 (n=1611)	0.793 (n=3591)	0.821 (n=3553)	0.827 (n=13620)
45-64	0.757 (n=587)	0.761 (n=5339)	0.8 (n=1567)	0.803 (n=24611)
65+	0.7095 (n=174)	0.724 (n=6658)	0.776 (n=665)	0.78 (n=48104)

Notes: Utility = combined physical and mental healthy days

Table 2. Utility scores by cigarette use, depression, and e-cigarette status - women ages 18+

	Depressed		Not depressed	
Age	Use E-cigarette	Not Use E-cigarette	Use E-cigarette	Not Use E-cigarette
Never smoking				
18-24	0.797 (n=1324)	0.799 (n=4927)	0.826 (n=1214)	0.839 (n=11063)
25-44	0.776 (n=902)	0.793 (n=16036)	0.821 (n=1010)	0.827 (n=47255)

45-64	0.757 (n=199)	0.767 (n=18166)	0.797 (n=287)	0.809 (n=62577)
65+	0.7405 (n=67)	0.706 (n=15620)	0.792 (n=170)	0.787 (n=80496)
Current smoking				
18-24	0.779 (n=343)	0.797 (n=228)	0.817 (n=151)	0.832 (n=151)
25-44	0.767 (n=1535)	0.771 (n=4221)	0.801 (n=1026)	0.811 (n=4532)
45-64	0.717 (n=1182)	0.717 (n=7178)	0.778 (n=971)	0.795 (n=10115)
65+	0.7075 (n=238)	0.7075 (n=3186)	0.7725 (n=362)	0.773 (n=8112)
Former smoking				
18-24	0.797 (n=404)	0.799 (n=259)	0.821 (n=219)	0.829 (n=201)
25-44	0.771 (n=2182)	0.781 (n=5184)	0.811 (n=1742)	0.821 (n=7719)
45-64	0.717 (n=1340)	0.761 (n=9995)	0.797 (n=1331)	0.8 (n=19635)
65+	0.706 (n=374)	0.7405 (n=10909)	0.776 (n=578)	0.776 (n=38860)
